# On-line hemodiafiltration did not induce an overproduction of oxidative stress and inflammatory cytokines in intensive care unit-acute kidney injury

**DOI:** 10.1186/s12882-017-0785-1

**Published:** 2017-12-22

**Authors:** Kada Klouche, Laurent Amigues, Marion Morena, Vincent Brunot, Anne Marie Dupuy, Audrey Jaussent, Marie Christine Picot, Noémie Besnard, Delphine Daubin, Jean Paul Cristol

**Affiliations:** 1 Intensive Care Medicine Department, University of Montpellier Lapeyronie Hospital, 371, Av Doyen Gaston Giraud, 34295 Montpellier, France; 2 Departments of Biochemistry, University of Montpellier, INSERM U1046, CNRS UMR 9214, 34295 Montpellier cedex 5, France; 3 Departments of Medical statistics, University of Montpellier, INSERM U1046, CNRS UMR 9214, 34295 Montpellier cedex 5, France; 4 Lapeyronie University Hospital. PhyMedExp, University of Montpellier, INSERM U1046, CNRS UMR 9214, 34295 Montpellier cedex 5, France

**Keywords:** Acute kidney injury, On-line Hemodiafiltration, Oxidative stress, Inflammatory cytokines, Anti-inflammatory cytokines, Egf, Vegf, Mcp-1

## Abstract

**Background:**

Though on-line intermittent hemodiafiltration (OL-IHDF) is a routine therapy for chronic dialysis patients, it is not yet widespread used in critically ill patients. This study was undergone to evaluate efficiency and tolerance of OL-IHDF and to appreciate inflammatory consequences of its use in intensive care unit (ICU)-acute kidney injury (AKI) patients.

**Methods:**

In this prospective cohort study conducted in a medical academic ICU in France, 30 AKI patients who underwent OL-IHDF were included. OL-HDF used an ultrapure water production: AQ 1250 line with double reverse osmosis, a generator 5008 with a 1.8m^2^ dialyzer with Polysulfone membrane (Fresenius Medical Care). Tolerance and efficiency of OL-IHDF were evaluated as well as its inflammatory risk by the measurement of plasma concentrations of proinflammatory (Interleukin 6, IL1β, IL8, Interferon γ) and anti-inflammatory (IL4, IL10) cytokines, Epidermal growth factor (EGF), Vascular Endothelial growth factor (VEGF) and Macrophage Chemoattractive Protein-1 (MCP-1) before and after sessions.

**Results:**

Intradialytic hypotensive events were observed during 27/203 OL-IHDF sessions accounting for a mal-tolerated session’s rate at 13.3%. Mean delivered urea Kt/V per session was 1.12 ± 0.27 with a percentage of reduction for urea, creatinine, β2-microglobulin and cystatine C at 61.6 ± 8.8%, 55.3 ± 6.7%, 51.5 ± 8.7% and 44.5 ± 9.8% respectively. Production of superoxide anion by leukocytes, mean levels of pro- and anti-inflammatory cytokines and plasmatic concentrations of EGF, VEGF and MCP-1 did not differ before and after OL-IHDF sessions. We observed however a significant decrease of mean TNFα plasmatic concentrations from 8.2 ± 5.8 to 4.8 ± 3.5 pg/ml at the end of OL-IHDF.

**Conclusions:**

OL-IHDF was not associated with an increase in pro and anti-inflammatory cytokines, oxidative stress or EGF, VEGF and MCP-1 in AKI patients and seems therefore a secure and feasible modality in ICUs.

## Background

In intensive care units (ICUs), around 5% of patients suffering from acute kidney injury (AKI) require renal replacement therapy (RRT) [1]. Intermittent conventional hemodialysis (IHD) and continuous venovenous hemo(dia)filtration are the 2 principal modalities used for RRT. However, the ideal renal replacement method for intensive care patients remains under scrutiny [2, 3]. It should combine the advantages of continuous RRT (CRRT) with those of IHD, be simple to implement and induce minimal work with limited cost. On-line dialysis fluids preparation may fit these conditions since it offers favorable technical possibilities and highly flexible procedures to apply various forms of cost-effective high efficiency hemodiafiltration (HDF) modalities in intermittent or sustained mode [4–7].

On-line HDF (OL-HDF) is a RRT based on cold sterilization of dialysis fluid to prepare the infusate which is readily administered into the extracorporeal bloodstream. It necessitates a fully microbiological integrity of on-line produced dialysis fluids. In chronic dialysis facilities, this technique became a routine and safe modality of RRT and now represents the most effective dialysis therapy [8–10]. A few reports exist about its use in ICUs and its routine application is mainly restricted to ICU facilities working with a trained nephrological team [4–7, 11–17]. The limited use of this technique by intensivists is largely related to the technical complexity with water treatment and HDF machines and to the potential infectious risk of on-line produced fluids infusion especially in septic patients who represent the majority of admissions in ICUs. We have previously reported our experience of OL-HDF use in ICU [11]. A regular bacteriological control of dialysis fluids showed that this technique is safe and well tolerated [11]. Whether OL-HDF modulates plasma cytokine concentration and oxidative stress production is still not investigated. The combined use of synthetic biocompatible membrane and ultrapure dialysis fluid may limit the additional inflammatory risk induced by OL-HDF. However, this potential acute inflammatory risk induced by OL intermittent HDF (OL-IHDF) which remains possible in ICU-AKI has never been evaluated. We designed therefore a study to determine whether OL-IHDF would induce an overproduction of oxidative stress, cytokines and growth factors in critically ill patients.

## Methods

This observational prospective study was carried out at the Medical ICU of Lapeyronie University Hospital at Montpellier and was approved by the Ethics Research Committee of our hospital; PHRC régional: N° 2006-A00510–51.

### Patients

Since 2004, we exclusively used OL-HDF besides continuous therapies to treat our critically ill patients. During one-year period, we consecutively enrolled all patients admitted to the ICU with AKI requiring RRT support and who underwent OL-IHDF. Exclusion criteria included pregnancy, age < 18 years old, previous chronic renal failure, and severe neutropenia. Epidemiological data and severity of patients assessed by the Simplified Acute Physiologic (SAPS) II [18] and Sepsis-related Organ Failure Assessment (SOFA) scores [19] were collected. Decisions regarding the initiation, management, and discontinuation of RRT were made by the referring physician according to the KDIGO recommendations [20]. The choice of RRT modality was depending on patient hemodynamic stability and was daily re-evaluated. Patients with hemodynamic instability or severe fluid overload were preferentially treated with continuous venovenous hemodiafiltration and with OL-IHDF when they had or recovered hemodynamic stability. Only OL-IHDF sessions were investigated. Outcome was assessed at ICU discharge.

### On-line intermittent hemodiafiltration

#### On-line intermittent hemodiafiltration: Description, disinfection procedures

OL-IHDF was performed using a RRT generator (Fresenius 5008, Fresenius Medical Care, Bad Homburg, Germany) with a standard 1.8 m^2^ Polysulfone hemodiafilter HF80 (Fresenius Medical Care, Bad Homburg, Germany). Countercurrent dialysate flow (QD) was routinely set at 500 mL/min, on-line infusate flow in pre-dilution mode (Qi) at 100 mL/min and blood flow (QB) at 300 mL/min. Dialysate and infusate temperature were adjusted to 36 °C and the sodium dialysate concentration at 145 mmol/L. The net-ultrafiltration rate was adapted to the hemodynamic parameters and extracellular volume status of each patient. Our ICU water production and distribution system was identical to that routinely used in chronic dialysis facilities performing on-line therapies [21] as previously described [11]. Dialysate and infusate purity has been also validated previously [9, 11, 22] and was ensured by regular endotoxin and microbiological testing. Vascular access was obtained through double lumen jugular catheter, with unfractionated heparin for anticoagulation whenever needed.

#### On-line intermittent hemodiafiltration: Clinical tolerance

OL-IHDF clinical tolerance was investigated by collecting the following intradialytic parameters at baseline and every 30 min: pulse, temperature, mean arterial pressure (MAP). A pyrogenic reaction was defined as the onset of objective chills and an increase in temperature of more than 1 °C in a patient who had no recorded signs or symptoms of infection before RRT [23]. An intradialytic hypotensive event was defined by a 20% reduction of MAP or by an initiation or/and increase in vasoconstrictive agents’ dose.

#### On-line intermittent hemodiafiltration solute control and inflammatory mediators’ evaluation

##### Biological plasma parameters

Blood solutes including urea, creatinine, β2-microglobulin (β_2_-M) and cystatine C (CyC) were routinely monitored at the beginning and the end of each OL-IHDF session. Blood samples were collected at the end of the treatment by standard stop-flow technique [24] and after the first hour of treatment, simultaneously at the arterial and venous ports after a temporary net ultrafiltration cessation. Urea, creatinine, β_2_-M and CyC removals per session were evaluated by the percentage of solute reduction ratios according to: RR = [(Cpre–Cpost)/Cpre]*100 where Cpre and Cpost are respectively pre-treatment (baseline) and post-treatment concentrations [25]. Kt/V were determined by using Daugirdas second generation, single pool urea kinetic model equation: Kt/V = −ln (T-0.008 * time duration session in minutes) + (4–3.5*T)*(UF/weight_postsession_) where T represents plasma urea_postsession_/urea_presession_ [26]. Instantaneous whole blood (K^W.B^) and plasma water solutes clearances (K^P.W.^) were estimated as follows: K^W.B^ = QB*[(Cart-Cven)/Cart] where QB is effective blood flow, Cart and Cven are solute concentration in arterial and venous blood line; K^P.W.^ = K^W.B^ *(1–0.00107*Tp)*[(SPC*Ht) + (1 - Ht)] where Ht is the patient’s predialysis hematocrit level, Tp the average of total protein level in arterial and venous blood line (Tp = [Tp_art_ + Tp_ven_]/2) and solute partition coefficient: 0.86 for urea, 0.73 for creatinine, and 0 for β_2_-M and CyC [27].

##### Determination of superoxide (O_2_^°-^) anion production by whole blood

O_2_^°-^ anion production was measured in blood samples before and after OL-IHDF sessions. It was determinated in 200 μL of fresh whole blood (treated immediately after collection) diluted in 820 μl of DMEM medium and 200 μL of lucigenin (1.5.10^−4^ mol/L) (Sigma Chemical, Saint Quentin Fallavier, France) [28]. After a 20-min incubation at 37 °C under gentle agitation, whole blood was stimulated by using Phorbol 12-Myristate 13-Acetate (PMA) (10^−7^ M) and the luminescence was immediately recorded at 37 °C by means of a Victor Wallac luminometer (Perkin Elmer, Turku, Finland). Luminescence intensity was normalized to leukocyte count. Response of PMA-free whole blood (basal O_2_^°-^ production) incubated simultaneously was used as control and considered as equal to 100%. To rule out autoproduction of O_2_^°-^ by lucigenin or by plasma compounds, O_2_^°-^ production was determined in whole blood, de-leukocyted blood, plasma and culture medium. Imprecision studies of O_2_°- production measure were as follows: intra-assay CV = 3.5% (basal O_2_°- production) and 3.9% (PMA-stimulated O_2_°- production); interassay CV = 5.0% (basal O_2_°- production) and 9.7% (PMA-stimulated O_2_°- production).

#### Determination of plasmatic cytokines, growth factors, and advanced oxidation protein products, measurements

Pre-and post-OL-IHDF sessions blood samples were immediately centrifuged at 1000 g for 10 min at 4 °C and stored at −80 °C until use. A panel of cytokines was determined on frozen plasma using a proteomic approach on an Evidence Investigator® biochip system (Randox, Mauguio, France). This proteomic method allows the simultaneous determination of IL_1β_, IL_4_, IL_6_, IL_8_, IL_10_, interferonγ, Epidermal growth factor (EGF), Vascular Endothelial growth factor (VEGF), Tumor Necrosis factor α (TNFα) and Macrophage Chemoattractive Protein-1 (MCP-1) levels. After addition of a sample (100 μl) to the biochip, the degree of binding of each analyte to its specific ligand is determined using a chemiluminescence light source and quantified using a super-cooled charge-coupled camera and an image-processing software [29].

Plasma Advanced Oxidation Protein Products (AOPP) levels (μM/l) were measured in pre- and post-OL-IHDF sessions blood samples by spectrophotometry [30, 31].

### Statistical analysis

Statistical analysis was performed using SAS Entreprise Guide version 4.1. We first performed a descriptive analysis by computing frequencies and percents for categorial data, means, standard deviations, quartiles and extreme values for continuous data. For every patient included, 2 or more OL-IHDF sessions were investigated. The session that induced the highest post treatment cytokine increase was solely analyzed per patient. We checked for normality of continuous data distribution (O_2_^°-^ anion production, cytokines and proinflammatory mediators measurements), using Shapiro-Wilk’s tests. To analyze differences between before and after treatment measurements, univariate analysis was performed using two-tailed Student t-test, or two-tailed Mann-Whitney-Wilcoxon’s test (signed Rank Statistic) when appropriate. Kinetic of oxidative stress and cytokine release before and after OL-IHDF was also investigated in all included sessions using a linear mixed model. A value of *p* < 0.05 was considered significant.

## Results

### Patient demographic data

During the study period, 34 of 51 patients admitted to our ICU for severe AKI treated by RRT were included in the study. Main reasons for non-inclusion were: contra-indication (8 patients for aplasia, 2 for non-consent, and 2 for life expectancy less than 48 h), and 5 patients treated only with continuous RRT. In addition, 4 patients were excluded because of missing data. Thus, 30 patients were enrolled in the study and completely analyzed. Age, gender, cause of AKI, severity scores are listed in Table 1. The cause of AKI was septic in approximately ¾ of the cases. All patients were anuric, treated by vasoactive agents and most of them ventilated. The ICU mortality rate was 26.7%.

**Table 1:** Epidemiological data of patients

Patient characteristics	*n* = 30
Age, years	61.1 ± 15.3
Male, n (%)	25 (83.3)
SAPS II	58.4 ± 20.8
APACHE II	29.8 ± 6.6
SOFA	11.6 ± 3.8
Mechanical ventilation, n (%)	19 (63.3)
Vasoactive support, n (%)	28 (93.3)
Causes of AKI, n (%)	
Septic	21 (70)
Ischemic	11 (36.6)
Toxic	12 (40)
Miscellaneous	4 (13.3)
ICU mortality, n (%)	8 (26.7)

All parameters, otherwise specified, are presented as mean ± standard deviation

Parameters, clinical and tolerance evaluation of on-line intermittent hemodiafiltration sessions.

Ol-IHDF duration time ranged from 4 to 6 h with a median time at 4.8 h. Sessions parameters were as follows: blood flow: 285(326–185) ml/mn, dialysate flow: 468 (442–489) ml/mn, predilution infusate flow: 89 (110–85) ml/mn with convection volume at 28 (20–36) l per session. Venous recirculation was less than 5% in all sessions.

Among the 203 OL-IHDF sessions (>3/patient) evaluated, mean arterial pressure (MAP) increased from 86.2 ± 16 to 90 ± 16 mmHg after treatment (*p* < 0.05) with a significant increase of MAP after 119/203 (58%) sessions. An intradialytic hypotensive event was observed during 27 sessions accounting for a mal-tolerated session’s rate at 13.3% (Table 2). Cessation of ultrafiltration was sufficient to restore hemodynamic stability in 23 sessions while a fluid challenge or a vasopressor support was necessary during the remaining sessions (Table 2). No pyrogenic reactions occurred among all OL-IHDF sessions performed.

**Table 2:** On-line IHDF sessions with hypotensive events

OL-IHDF sessions, *n* = 203	n (%)
*Sessions with hypotension*	27 (13.3)
Requiring only UF cessation	23 (11.3)
Requiring a vasopressor support	5 (2.5)
Requiring a fluid challenge	4 (2)
Requiring a dialysis cessation	0 (0)

One or more therapeutic interventions may be used

### On-line intermittent hemodiafiltration efficiency and solutes control

Evaluation for efficiency and inflammatory consequences of OL-IHDF was achieved for 76 out of 203 (37.4%) OL-IHDF sessions accounting for at least one session per patient (choosing the session with the highest cytokine increase after treatment according to Statistical analysis). Mean delivered urea Kt/V session was 1.12 ± 0.27. RR for urea, creatinine, β2-M and CyC were respectively 61.6 ± 8.8%, 55.3 ± 6.7%, 51.5 ± 8.7% and 44.5 ± 9.8%. K^W.B.^ and K^P.W.^ were 239.2 ± 22.3 and 213.4 ± 20.7 mL/min for urea, 197 ± 22 and 168.6 ± 20.5 mL/min for creatinine, 58.9 ± 17.3 and 38.5 ± 10.9 mL/min for CyC, 77.8 ± 29.6 and 50.3 ± 17.4 mL/min for β2-M. Albuminemia increased from 27.5 ± 4.0 g/L to 28.5 ± 4.3 g/L (*p* < 0.05).

### On-line intermittent hemodiafiltration and inflammatory mediators

**Fig. 1:**
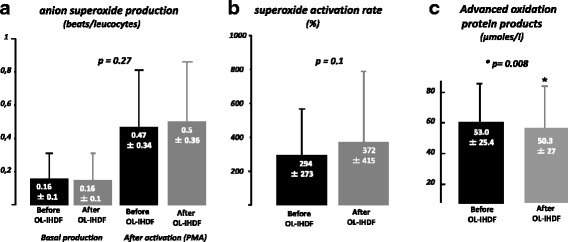
Anion superoxide production (**a**), superoxide activation rate (**b**), and advanced oxidation protein products plasmatic levels (**c**) before and after OL-IHDF sessions. Values are shown as mean and standard deviation. Phorbol 12-myristate 13-acetate (PMA), On-line intermittent hemodiafiltration (OL-IHDF)

Mean basal and PMA-stimulated production of O_2_°- anion by leukocytes did not differ before and after OL-IHDF sessions (Fig. 1a). The activation rate of PMA-stimulated O_2_°- production reached 294% ± 273 at the initiation and 372% ± 415 at the end of OL-IHDF, but differences were not statistically significant (Fig. 1b). However, OL-IHDF sessions provided a slight but significant decrease in AOPP (*p* = 0.008) (Fig. 1c).

**Fig. 2:**
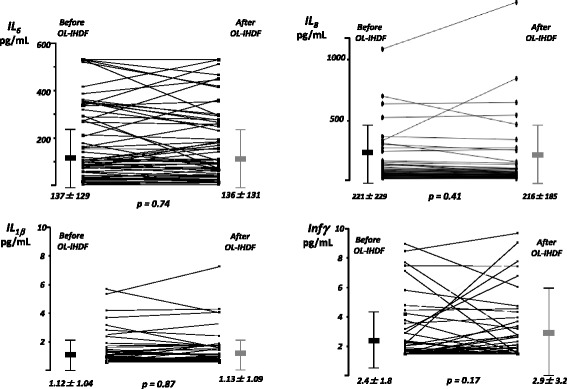
Mean plasmatic concentrations of pro-inflammatory cytokines before and after OL-IHDF sessions. Values are shown as mean and standard deviation. On-line intermittent hemodiafiltration (OL-IHDF), Interleukin 6 (IL_6_), Interleukin 8 (IL_8_), Interferon γ (infγ), Interleukin 1β (IL_1β_)

**Fig. 3:**
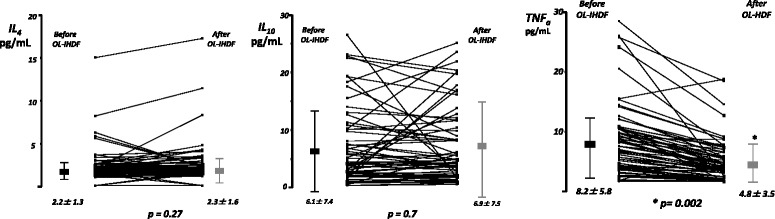
Mean plasmatic concentrations of anti-inflammatory cytokines and TNFα before and after OL-IHDF sessions. Values are shown as mean and standard deviation. Interleukin 4 (IL_4_), Interleukin 10 (IL_10_), Tumor Necrosis Factor alpha (TNFα)

**Fig. 4:**
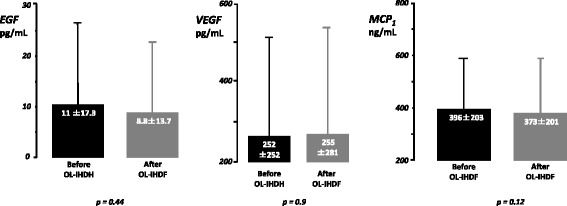
Mean plasmatic concentrations of Endothelial Growth factor (EGF), Vascular Endothelial Growth factor (VEGF), Macrophage Chemoattractive protein 1 (MCP-1) before and after OL-IHDF sessions. Values are shown as mean and standard deviation

Cytokines plasmatic measurements showed a significant heterogeneity between individuals before initiation of OL-IHDF but their variations after OL-IHDF sessions remained totally similar. Thus, mean levels of pro- (IL_6_, IL_10_, IL_8_, interferon γ) and anti- (IL_4_, IL_10_) inflammatory cytokines were not significantly different before and after OL-IHDF sessions (Figs. 2 and 3). We observed however a significant decrease of mean TNF_α_ plasmatic concentrations from 8.2 ± 5.8 to 4.8 ± 3.5 pg/mL after OL-IHDF sessions, a reduction ratio at 41.4% (Fig. 3). Analysis of plasmatic concentrations of EGF, VEGF and MCP-1, before and after OL-IHDF sessions showed no significant differences (Fig. 4).

## Discussion

The present study demonstrated that OL-IHDF achieved an adequate dialysis dose with a fair hemodynamic tolerance in critically ill patients, and was not associated with an increased inflammatory risk. It did not induce an overproduction of oxidative stress and of pro- and anti-inflammatory cytokines but a significant decrease of TNF_α_ and AOPP plasmatic levels.

On-line substitution fluid preparation is a standard feature of modern dialysis devices for chronic treatments [32]. In ICU settings, on-line RRT modalities are however rarely used and their routine application is mainly restricted to ICU facilities working with a trained nephrological team [4–7, 9, 11–17]. The carefulness of intensivist to use OL-IHDF is related to the potential infectious risk and deleterious effects of on-line produced fluid infusion especially in septic patients who represent the majority of those admitted to ICUs. On-line preparation is not accompanied by on-line control of the microbiological quality and inadequate infusate would be administered directly without prior product check and release. In a previous work, we have evaluated, over a 7 year-period, the purity of on-line produced ultrapure water and dialysis fluids by a weekly bacterial control monitoring in ICU settings: more than 90% of samples showed negative bacterial growth and undetectable levels of endotoxin indicating an overall compliance rate of 99% [11]. However, water and dialysis fluids may still contained cytokines-inducing substances other than endotoxins like breakdown products of microorganisms, peptidoglycans and β-glycans [33]. They have the potential capability of penetrating ultrafiltration and dialysis membranes with subsequent blood exposure and intradialytic cytokine and oxidative stress induction [34]. We aimed therefore to assess the inflammatory risk for critically ill patients receiving OL-HDF by the quantification of cytokines, oxidative stress and growth factors potentially produced during OL-IHDF.

Critically ill patients with AKI have higher circulating plasma concentration of inflammatory biomarkers implicated in RRT dependence and mortality than those without AKI [35, 36]. This inflammatory process is partly due to the generation of oxidative stress which is mainly of multifactorial origin including sepsis, and accumulation of uremic toxins in case of AKI but may be also related to RRT modalities. Indeed, online therapy itself can exacerbate oxidative stress production through leukocytes activation induced by dialysis and substitution fluids. Herein, we found that OL-IHDF did not alter superoxide anion production by leukocytes either basal or after stimulation by PMA. Our AKI patients treated by RRT have an increased levels of AOPPs [37–39], varying from 35 to 120 μmol/L as previously reported by Du et al. [39] but lower than those observed by Lentini et al. [38]. We observed that OL-IHDF provided a slight but still significant decrease in AOPP plasma concentrations. Of note, it has been suggested earlier that AOPPs contribute to the progression of renal failure and that higher AOPP levels are associated with poor renal recovery [40]. Cytokine induction has been also considered as the trigger of the inflammatory response and a critical parameter of dialysis biocompatibility during RRT [41]. Moreover, high levels of pro-inflammatory cytokines have been associated with increased mortality in AKI [35, 42]. We found that both pro- and anti-inflammatory cytokines plasma levels did not increase after OL-IHDF suggesting that it might not alter the balance of cytokines production. Other studies reported, like us, the lack of cytokines reduction by hemodiafiltration [43]. Substance clearance is dependent on its molecular size but also on ultrafiltration rate and on whether the substitution fluid is administered before and after the filter. In our study, we used a predilution modality of HDF diluting the blood before filter passage and convection volumes were less than 30 l per session explaining, at least for a part, the observed non significative reduction of plasmatic cytokines.

Last, OL-IHDF sessions were not associated with an increased production of VGEF, EGF and MCP-1 in our patients. Enhanced production of growth factors after endothelial activation has been reported in critical conditions especially sepsis-related AKI leading to increased capillary permeability, systemic vasodilatation and multi-organ failure [44, 45]. Chancharoenthana W [46] reported however that OL-HDF provided a significant removal of VEGF and was associated with better renal outcome as compared to high-flux hemodialysis.

Furthermore, on-line IHDF was best tolerated and achieved an adequate urea reduction rate. We did not observe any pyrogenic reactions whereas data on chronic on-line HDF reported an incidence at 0.04% [47]. A low incidence of intradialytic hypotensive events was also repertoried (13.3%) lower than recently reported (18.7%) (47). However, we may not state that OL-IHDF lead to a better hemodynamic tolerance than other modalities especially continuous therapies since this study was only observational.

We must acknowledge some limitations to the study. First, our work shares the limitations of single-center studies. Our unit is indeed familiar with online therapies which are not the general rule in ICU settings. Second, the number of patients included may be considered small but a high number of OL-HDF sessions were analyzed and secure our observations. Third, our RRT patients exhibited a cellular reactivity as reflected by inflammatory and oxidative stress parameters with a medium intensity thanks to the biocompatibility membranes. An evaluation of more sensitive biomarkers like isoprostanes may evaluate more thoroughly oxidative stress. Nevertheless, most of the tested parameters were not modified during the on-line sessions performed, a result that represents our primary hypothesis in this work. Last, outcome was not studied in this study since our concern was focused on feasibility and potential risk of this technique in critically ill patients.

## Conclusions

In conclusion, our study shows that OL-IHDF does not induce additional inflammatory risks in critically ill patients with AKI and may be used securely in these settings. On-line production of ultrapure water seems to be very useful for ICU acute renal failure as it gives possibilities for a large scale of dialysate and infusate rate prescription. Further studies should however investigate its effect on all-cause mortality in comparison to other RRT modalities.

## Abbreviations

AKI: Acute kidney injury
Cpost: Post-treatment concentration
Cpre: Pre-treatment concentration
CRRT: Continuous renal replacement therapy
CyC: Cystatine C
EGF: Epidermal growth factor
HDF: Hemodiafiltration
ICU: Intensive care unit
IHD: Intermittent conventional hemodialysis
K^P.W.^: Plasma water solutes clearances
Kt/V: Clearance adjusted for total body volume
K^W.B.^: Instantaneous whole blood solutes clearances
MCP-1: Macrophage Chemoattractive Protein-1
O2°-: Superoxide anion
OL-HDF: On-line hemodiafiltration
OL-IHDF: On-line intermittent hemodiafiltration
PMA: Phorbol 12-myristate 13-acetate
QB: Blood flow
QD: Dialysate flow
Qi: Infusate flow
RR: reduction ratio
RRT: Renal replacement therapy
SAPS II: Simplified Acute Physiologic II score
SOFA: Sepsis-related Organ Failure Assessment score
TNF α: Tumor Necrosis factor alpha
VEGF: Vascular Endothelial growth factor
β2-M: β2-microglobulin
